# Comparison of isolated venous approach with the standard approach in children undergoing patent ductus arteriosus device closure

**DOI:** 10.1186/s43044-020-00100-1

**Published:** 2020-09-29

**Authors:** Lipi Uppal, Manoj Kumar Rohit, Parag Barwad, Sanjeev Naganur, Uma Debi, Ganesh Kasinadhuni, Krishna Santosh, Pruthvi CR, Saroj Sahoo

**Affiliations:** 1 Department of Cardiology, Post Graduate Institute of Medical Education and Research, Chandigarh, 160012 India; 2 Department of Radiodiagnosis, Post Graduate Institute of Medical Education and Research, Chandigarh, 160012 India

**Keywords:** Venous alone access, PDA device closure, Arterial thrombosis

## Abstract

**Background:**

Transcatheter device closure is a safe procedure recommended in children with patent ductus arteriosus (PDA). While the standard procedure uses arterial and venous femoral access, it poses risk of vascular complications especially in young infants. Isolated venous approach has been tried in a few studies and was found to be non-inferior to the standard technique. In this prospective observational study, we have compared the two vascular approaches of PDA device closure in pediatric patients and have also studied the feasibility of this approach in young children with weight < 6 kg.

**Results:**

PDA device occlusion was performed with either one of the approaches—venous alone (group I) or standard approach (group II) in a total of 135 children enrolled prospectively. The baseline data, procedural outcomes, vascular complications, and radiation dose were compared between the two groups.

Fifty-two and 83 children were included in group I and group II, respectively. A total of 22 children (16%) (13 in group I; 9 in group II) had weight < 6 kg. In group II, 6 children (7.2%) had vascular site complications treated with heparin infusion with two children requiring thrombolysis. Another child in group II developed intravascular hemolysis following residual shunt, requiring surgical device retrieval and closure. No significant differences were observed in mean fluoroscopic time (*p* = 0.472) and air kerma between the two groups (*p* = 0.989).

**Conclusion:**

Transcatheter PDA device closure without arterial access is a feasible and safe option in children including young infants. This technique avoids the risk of vascular complications although requires careful case selection.

## Background

Patent ductus arteriosus (PDA) is one of the most common congenital diseases representing 5–10% of all congenital heart lesions [1]. Percutaneous transcatheter closure has become the mainstay of treatment of symptomatic PDA since its initial description by Porstmann et al. in 1967 [2]. Traditionally, the device closure of PDA requires an arterial access for delineating the size and anatomy of PDA, while device deployment is performed via the venous route. However, arterial access may be associated with vascular complications, the rates of which have ranged from 3.8 to 16% in various studies [3, 4]. This risk is further compounded in younger children with small caliber vessels and may have short- and long-term implications [4]. Apart from vascular complications, the use of arterial access is associated with longer fluoroscopic time, contrast volume, and longer hospital stays [5]. The aim of this study was to report experience of PDA device closure using isolated venous access in a large group of patients and to compare outcomes with the standard approach requiring both venous and arterial access.

## Methods

### Study design and patient selection

This was a prospective observational study, conducted over a period of 1 year and 4 months from July 2017 to November 2018 in a tertiary care hospital in North India. A total of 135 patients were enrolled. The inclusion criteria included patients with age between 4 months and 16 years and patients with clinical and echocardiography evidence of PDA. Patients with severe pulmonary hypertension, other associated congenital heart diseases, and “silent ductus” were excluded. The study was approved by the “Institute Ethics Committee.”

### Procedural strategy

After recording the basic data, a detailed echocardiographic evaluation was performed for all patients using Philips EPIQ 7 (Philips healthcare^TM^, Amsterdam, Netherlands) as per institute protocol. PDA was assessed in high parasternal and suprasternal long axis views with its size corresponding to the measurement obtained at the pulmonary end of the ductus.

**Fig. 1:**
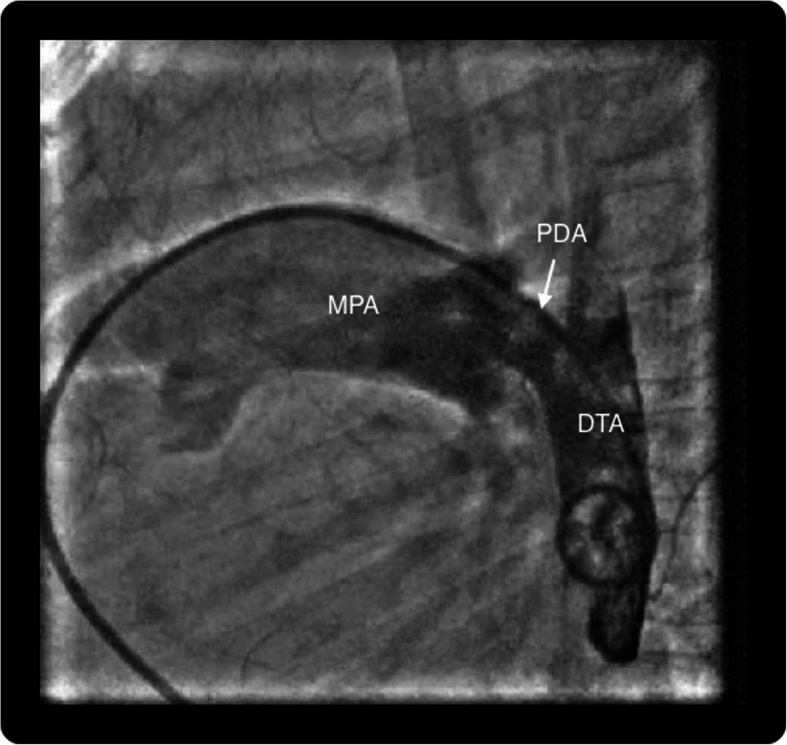
Contrast injection through 5-Fr pigtail catheter in lateral projection inserted from the pulmonary artery to the descending aorta across patent ductus arteriosus. MPA main pulmonary artery, DTA descending thoracic aorta

**Fig. 2:**
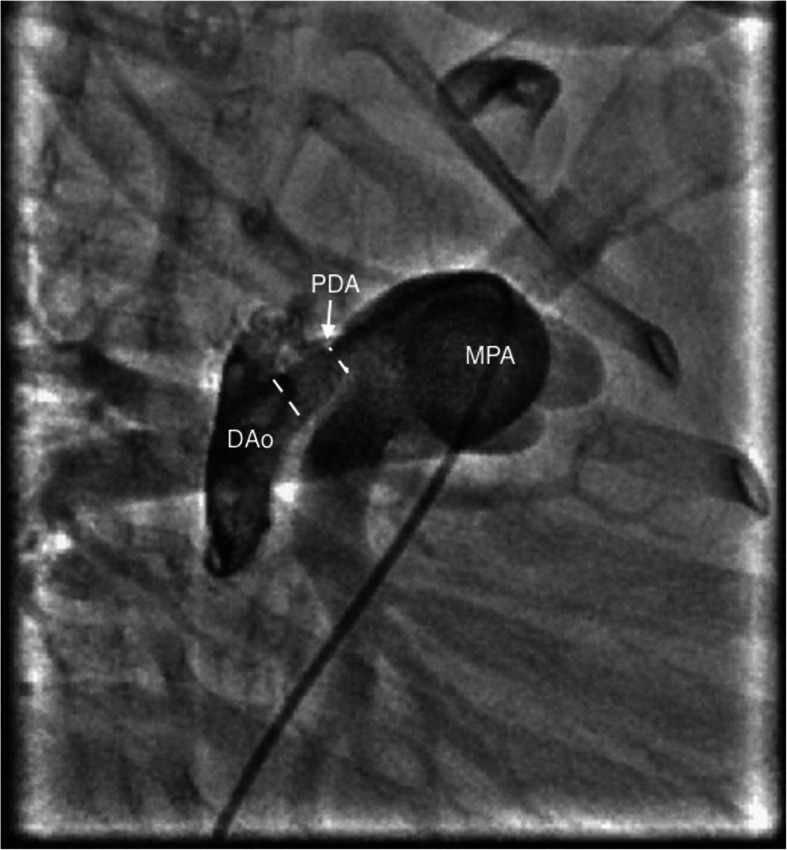
Contrast injection in right anterior oblique (RAO) view 30° with pigtail catheter crossing from the main pulmonary artery to the descending aorta depicting conical-shaped PDA

**Fig. 3:**
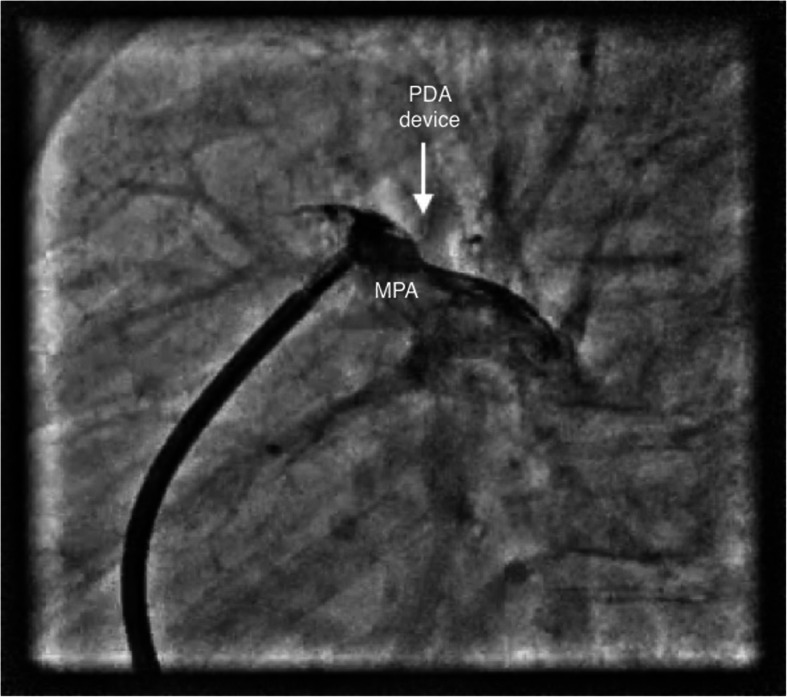
The position of the PDA device is ascertained by continuous hand injection via the long delivery sheath filling pulmonary artery

All the procedures were performed by 3 independent pediatric cardiologists, and the choice of access was left to their discretion. A written informed consent was obtained from the parents following which all procedures were performed under conscious sedation. Children were divided into two groups, group I: those who underwent the procedure with venous access alone and group II: patients with both arterial and venous access. In group I, the size and anatomy of PDA were assessed by contrast injection via pigtail negotiated into the descending aorta across PDA from the pulmonary artery. The angiographic view was taken in the left lateral view 90° (Fig. 1), and additional right anterior oblique (RAO) 45° (Fig. 2) was used if the former alone could not profile the duct appropriately. With pulmonary end as the reference, the device was upsized by 2 mm and correlated with echocardiographic measurements. The device was deployed from the venous end with guidance of contrast injections, when necessary (Fig. 3). 2D echocardiography was used for confirmation of the precise position of the device and any residual flow across the device. Pulmonary artery pressures were measured from the sidearm of the long delivery sheath before releasing the device.

In group II children, fluoroscopic sizing of PDA was done from aortic angiogram obtained via the femoral artery. The device was deployed from the venous end. Its position and residual flow were confirmed on repeat angiogram. Aortic gradient across the device was measured in large devices to look for any iatrogenic coarctation.

All the children were given 100 IU/kg of heparin during the procedure, and manual compression was used to achieve hemostasis. The radiation doses were analyzed using total fluoroscopic time and air kerma for all the patients. Success was defined as a closed duct with procedure free from serious complications like death, device embolization, residual shunt, and device protrusion into adjacent structures requiring reintervention.

The following devices were used in our study:

1. Amplatzer duct occluder (ADO) (AGA Medical Corporation, Golden Valley, MN)
2. Cocoon duct occluder (Vascular Innovations, Thailand)
3. Lifetech duct occluder (Lifetech Scientific Inc, Shenzhen, China)

### Follow-up

Following the procedure, all the children were monitored for clinical signs of limb ischemia (bilateral femoral pulses, temperature, color). All children in group II underwent a Doppler ultrasound of the local site. 2D echocardiography for residual flow across the device, aortic impingement, and pulmonary artery encroachment of the device was performed at 24 h and at 1-month follow-up.

### Statistical analysis

All the data was collected prospectively. Statistical Package for Social Sciences (SPSS Inc. version 16.0 for Windows) was used for the analysis of data. All quantitative or continuous variables were estimated using measures of central tendency (mean) and measures of central dispersion (standard deviation). Normality of data was verified by measures of Kolmogorov-Smirnov tests of normality. For group comparisons of categorical variables, chi-square test or Fischer’s exact test was used. Group comparisons for skewed data were done by Mann-Whitney for 2 groups. For normally distributed data, the Student *t* test was used.

## Results

A total of 135 children with clinically significant PDA between the ages of 4 months and 16 years were enrolled during the study period. The isolated venous approach was used in 59 patients, and the standard approach was used in 76 patients. Out of 59 patients undergoing the isolated venous approach, 7 patients required arterial access in view of insufficient visualization of the anatomy of PDA (4 patients), to measure aortic gradient across the large device being deployed (2 patients) and large residual shunt on echocardiography (1 patient). Thus, a total of 52 patients were included in group I and 83 patients were included in group II for final statistical analysis.

### Baseline characteristics (Table 1)

Mean age and weight of the children in group 1 was significantly less than the mean age and weight of children in group II [38.94 (± 48.1) vs 60.11 (± 60.3) months, *p* = 0.017 and 14.4 (± 11.2) vs 18.9 (± 14.6) kg, *p* = 0.043]. This was probably because of the preferential venous approach used in younger children in the study. Females constituted 50% and 55% of the study patients in group I and group II, respectively. Mean PDA diameter was similar in both groups (4.3 ± 2.6 vs 5.1 ± 3.2 mm, *p* = 0.634). Operator 1 performed 68 procedures (50%), operator 2 performed 27 procedures (20%), and operator 3 performed 40 procedures (30%). We used Amplatzer duct occluder (52 patients), Cocoon duct occluder (61 patients), and Lifetech duct occluder (21 patients) in our study.

**Table 1:** Baseline characteristics in the two groups of children with PDA device closure

Variables		Group I (*n* = 52)	Group II (*n* = 83)	*p* value
Age (months) ± SD		38.94 ± 48.1	60.11 ± 60.3	0.017*
Sex	Males	26 (50%)	37 (44.5%)	0.539
	Females	26 (50%)	46 (55.4%)	0.662
Weight (kg)	Mean ± SD	14.4 ± 11.2	18.9 ± 14.6	0.043*
	≤ 6.0 kg	13 (25%)	9 (10.8%)	0.030*
	> 6.0 kg	39 (75%)	74 (89.2%)	
PDA size (mm)	Mean ± SD	4.3 ± 2.6	5.1 ± 3.2	0.634

*represents significant value *p* <0.05

### Complications (Table 2)

All vascular complications were seen in group II of the study. A total of 8 patients (9.6%) had local site hematoma which was managed with manual compression, and none of the patients had life-threatening bleed. Another 6 patients (7.2%) had absent femoral pulse and the limb was differentially cold as compared to the contralateral limb, 4 h after the procedure. They were preemptively started on intravenous heparin infusion at 25 IU/kg/h with further titration according to activated partial thromboplastin time. Doppler ultrasound revealed evidence of complete or partial thrombosis in four out of these six children, while the remaining two had normal triphasic flow. Flow restoration was possible in two of the four children with unfractionated heparin infusion alone given for 12–24 h. The remaining two were thrombolyzed with intravenous streptokinase (bolus dose of 4000 IU/kg followed by infusion at 2000 IU/kg/h) in view of worsening limb ischemia in form of color changes and mottling. Three days of heparin treatment was completed in them, and there was normal flow on follow-up Doppler ultrasound.

**Table 2:** Outcomes of the procedure in the two groups

Procedural outcomes		Group I	Group II	*p* value
Mean fluoroscopic time (± SD) (minutes)		4.9 (± 2.5)	5.3 (± 3.0)	0.472
Mean air kerma (± SD) (mGy)		10.54 (± 10.2)	10.51 (± 14.8)	0.989
Major complications	Total	*N* = 0	*N* = 7	0.032*
	Hemolysis	0 (0%)	1 (1.2%)	
	Femoral artery thrombosis	0 (0%)	4 (4.8%)	
	Transient pulselessness	0 (0%)	2 (2.4%)	
Minor complications	Total	*N* = 5	*N* = 9	0.820
	DTA flow acceleration	4 (7.6%)	1 (1.2%)	
	LPA flow acceleration	1 (1.9%)	1 (1.2%)	
	Local site hematoma	0 (0%)	8 (9.6%)	
Closure time	Immediately	43 (82.7%)	64 (77.1%)	0.536
	Up to 24 h	8 (15.4%)	15 (18.1%)	0.686
	> 24 h	1 (1.9%)	2 (2.4%)	0.852
Operator mean air kerma (± SD) (mGy)	1	6.27 (± 3.41)	8.67 (± 2.64)	0.002*
	2	7.00 (± 6.08)	6.79 (± 2.46)	0.989
	3	15.8 (± 13.13)	20.2 (± 31.19)	0.540
Operator mean fluoroscopic time (± SD) (minutes)	1	3.8 (± 1.30)	4.76 (± 1.52)	0.013*
	2	3.27 (± 1.58)	4.2 (± 2.05)	0.443
	3	6.5 (± 2.8)	8.3 (± 4.7)	0.138

*represents significant value *p* <0.05

The children exhibiting normal color flow on Doppler had their heparin stopped consequently with the provisional diagnosis of arterial spasm, recanalized thrombus, or tight compression bandage.

Closure occurred immediately in 43 patients (82.7%) of group I vs 64 (77.1%) of group II (*p* = 0.536). In one child of group II, the procedure was abandoned due to large residual shunt even after using the device of maximum size. Another child in group II who had residual shunt developed hemolysis on day 2 of the procedure after deployment of 12/14mm Cocoon duct occluder. PDA device was subsequently retrieved during open repair along with ductal ligation. None of the other patients had residual flow at a 1-month follow-up.

A total of 4 patients in group I had mild gradient (15 to 20 mm Hg) across the device in the aorta and 2 patients, one from each group, had mild gradient (17 mm Hg each) across the left pulmonary artery. There was no significant difference noted in total air kerma (*p* = 0.472) and mean fluoroscopy time (*p* = 0.989) between the two groups. However, on subgroup analysis, operator 1 had a significant reduction of radiation dose and fluoroscopic time in group I vs group II (6.27 ± 3.41 mGy vs 8.67 ± 2.64 mGy; *p* = 0.002 and 3.8 ± 1.30 min vs 4.76 ± 1.52 min; *p* = 0.013, respectively). There was no significant difference between the two groups for the other 2 operators (Table 2).

## Discussion

PDA device closure is a relatively safe procedure barring from its rare complications of device embolization, hemolysis, and encroachment on surrounding structures including the left pulmonary artery and descending aorta [6]. Most of the studies report a high incidence of success rates, with successful device closure seen in 97–100% of the cases [7, 8]. However, as the procedure is increasingly being used in younger infants and neonates, there is a growing concern for procedure-related complications especially access site vascular injury which can have devastating consequences [9]. The use of smaller sheaths and catheters in this regard has shown to reduce the incidence of arterial thrombosis; however, the risk is not totally eliminated till arterial cannulation is used for the procedure [10]. This observational study was aimed to assess the feasibility of isolated venous access in children requiring PDA device closure, when compared to the traditional method of using both arterial and venous routes.

Arterial access during PDA device closure assists in angiographic profiling of the size and anatomy of PDA. After deployment of the device, it helps in assessing the residual shunt and any significant gradient across the aorta. Arterial access may also be required in cases of hemodynamic collapse, device embolization, and patients with pulmonary hypertension. Various studies however have reported a high incidence of vascular complications which includes arterial thrombosis, retroperitoneal bleeding, vessel tear, and dissection ranging from 2.5 to 3.8% with arterial access in pediatric catheterizations [4]. This risk is even higher for young children undergoing prolonged interventional procedures and is seen in up to 16% of infants on Doppler imaging [3].

Although the use of a single venous approach has been observed to produce successful outcomes, one of the main concerns remains that the crossing of PDA for initial sizing may induce ductal spasm and consequent undersizing of the device [11)]. Lack of precise size and morphological assessment can make the procedure difficult with frequent requirement of upsizing of devices. Careful preprocedural echocardiography thus becomes a prerequisite before embarking on only venous approach. Isolated venous access may also preclude from recognizing the iatrogenic coarctation of the aorta and measuring aortic gradient in large devices. Large PDA may similarly require arterial access for continuous pressure monitoring. Hence, previous studies also report the use of a successful venous approach in patients with careful preprocedure screening, although simultaneous arterial access may be required at different stages during the procedure [12, 13].

In a retrospective analysis by Gaurav et al., PDA device closure via the isolated venous approach could be successfully performed in 84% (151/179) of the patients without any vascular complications [12]. Liu et al. [14] similarly demonstrated successful PDA closure via only venous approach. No vascular complications were encountered, and the procedure was deemed safe for larger use including in infants.

We could successfully use the venous technique in 88% of the assigned patients as 7 patients required additional arterial access after initial attempt at isolated venous access.

The presentation of iatrogenic vascular complication immediately postprocedure includes acute limb ischemia or excessive bleeding, while long-term implications can lead to claudication and shortened length of the affected limb [14]. In the arterial access group (group II) in our study, access site complications were observed in 7.2% (*n* = 6, in 83 patients) vs none in the venous access group (group I). All these patients were less than 1 year of age with weight < 8 kg. Complications included common femoral artery thrombosis requiring intravenous heparin alone or thrombolysis in cases of a threatened limb.

The mainstay of treatment of acute limb ischemia involves parenteral anticoagulation, where heparin infusion alone is shown to be effective in 40% of the patients and, combined with streptokinase, complete revascularization can be achieved in 90% the cases [15]. Surgery can be offered in resistant cases for removal of the thrombus. We could successfully restore the blood flow by the use of heparin and/or streptokinase in all the patients within 24 h. All the patients had resolution of thrombus on follow-up ultrasound. Although rare, the use of fibrinolytics may be associated with fatal intracranial or gastrointestinal bleeding [16]; however, no excessive bleed was observed in any of the patient. One patient in our study developed hemolysis along with residual shunt and underwent surgical device retrieval. This is in accordance with previous studies such as by Jang et al. where major complications of hemolysis, infective endocarditis, and embolization were seen in 3.4%, and minor complications in 5.1% of the children undergoing PDA device occlusion [17].

In a randomized controlled trial by Thanopoulas et al. [18], in young children 2–24 months of age, 112 children were randomized for either venous only or standard access approach. Arterial complications were observed in 16% of the cases undergoing device closure via standard technique. The higher rates of arterial complications were probably secondary to younger population included in this study. We had 22 (16%) patients with weight < 6 kg. While 13 of them underwent PDA device closure by only venous access, the remaining 9 underwent the standard procedure. No significant complications were encountered in this population, and we could perform the device placement successfully for all the patients.

On subgroup analysis, fluoroscopy time and radiation dose were significantly reduced in group I in our study within cases of a single operator. This was likely as we had relied more on echocardiographic images to rule out aortic obstruction and residual shunts. The isolated venous approach may also facilitate early discharges due to less number of vascular complications. Previous studies have also demonstrated shorter procedural and fluoroscopic time due to a restricted number of cine angiograms [5]. Baykan et al. found increased fluoroscopic time in their cohort of patients since they used the return phase of the contrast angiography to see for adequacy of ductal closure [19].

The long-term complete occlusion with ADO devices is excellent with immediate closure rates of 66% and up to 97% at 1 month [20]. In Cocoon devices, long-term closure rates have been reported to be around 91.3% [21]. In our study, in majority of cases, Cocoon (45%) and ADO I (38%) devices were used and 80% of the children achieved immediate closure. None of the patients had residual shunt at 1 month of follow-up.

It was a single-center prospective experience where the decision to undergo only venous vs standard approach was decided by the primary operator. Due to state finance insurance schemes, devices used for PDA closure varied for different patients. Although the fluoroscopic time and radiation dose were reduced in only the venous approach, it can be secondary to less complicated cases chosen for the same.

## Conclusion

The study concludes that the isolated venous approach is a pragmatic approach for PDA device closure.

It requires however appropriate case selection with careful preprocedure and intraprocedural echocardiographic guidance.

## Data Availability

The data sets used and/or analyzed during the current study are available from the corresponding author on reasonable request.

## Abbreviations

PDA: Patent ductus arteriosus
LPA: Left pulmonary artery
DTA: Descending thoracic aorta
ADO: Amplatzer duct occluder
RAO: Right anterior oblique
